# Specific impact of β5t on proteasome subunit composition in cortical thymic epithelial cells

**DOI:** 10.1016/j.celrep.2021.109657

**Published:** 2021-09-07

**Authors:** Izumi Ohigashi, Yousuke Takahama

**Affiliations:** 1 Division of Experimental Immunology, Institute of Advanced Medical Sciences, University of Tokushima, Tokushima 770-8503, Japan; 2 Thymus Biology Section, Experimental Immunology Branch, National Cancer Institute, National Institutes of Health, Bethesda, MD 20892, USA; 3 Lead contact

## Abstract

β5t is a cortical thymic epithelial cell (cTEC)-specific component of the thymoproteasome, which is essential for the optimal production of functionally competent CD8^+^ T cells. Our recent analysis showed a specific impact of β5t on proteasome subunit composition in cTECs, supporting the possibility that the thymoproteasome optimizes CD8^+^ T cell development through the production of MHC-I-associated unique self-peptides in cTECs. However, a recent article reports that β5t regulates the expression of hundreds of cTEC genes and affects both CD4^+^ and CD8^+^ thymocytes by causing oxidative stress in thymocytes. The authors further analyze our published data and describe that they confirm their conclusions. Here, we examine the issues that they raise and conclude that, rather than regulating hundreds of genes in cTECs, β5t has a highly specific impact in cTECs on proteasome subunit composition. This Matters Arising Response article addresses the Apavaloaei et al. (2021) Matters Arising paper, published concurrently in *Cell Reports*.

## In brief

Ohigashi and Takahama report that β5t, encoded by *Psmb11*, has a highly specific impact in cortical thymic epithelial cells (cTECs) on proteasome subunit composition, rather than pervasively regulating the expression of hundreds of genes in cTECs.

## Graphical abstract

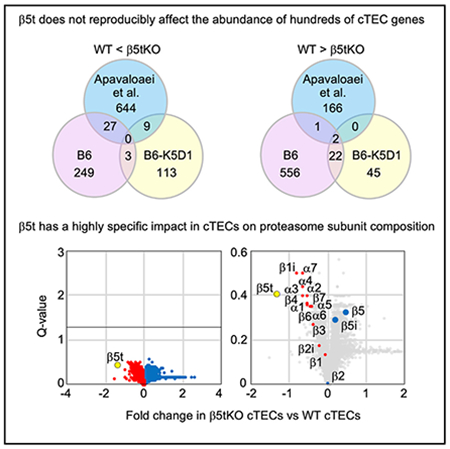

## INTRODUCTION

β5t, encoded by *Psmb11*, is a cortical thymic epithelial cell (cTEC)-specific component of the thymoproteasome, which is essential for the optimal production of functionally competent CD8^+^ T cells (Murata et al., 2007; Nitta et al., 2010; Xing et al., 2013; Takada et al., 2015). It has been suggested that β5t-containing thymoproteasomes produce a cTEC-specific set of major histocompatibility complex (MHC) class I-associated self-peptides, which govern the positive selection of CD8^+^ T cells in the thymic cortex (Sasaki et al., 2015; Murata et al., 2018; Ohigashi et al., 2021). Our recent trans-omics (i.e., multi-layer combination of proteomics and transcriptomics) analysis of isolated TEC subpopulations revealed a highly specific impact of β5t on proteasome subunit composition in cTECs, supporting the possibility that the β5t-containing thymoproteasome governs CD8^+^ T cell development through the proteasomal production of MHC class I-associated unique self-peptides in cTECs (Ohigashi et al., 2019).

A recent article by Apavaloaei et al. (2019) reported that β5t regulates the expression of hundreds of cTEC genes that modulate lymphostromal interactions primarily via the Wnt signaling pathway. It was reported that cTECs from β5t-deficient mice acquire features of medullary TECs (mTECs) and retain CD8^+^ thymocytes in the thymic cortex, thereby impairing thymocytepositive selection and causing dramatic oxidative stress in both CD4^+^ and CD8^+^ thymocytes, leading to apoptosis of CD8^+^ thymocytes (Apavaloaei et al., 2019). The authors concluded that β5t pervasively affects both CD4^+^ and CD8^+^ thymocytes through the regulation of gene expression in cTECs (Apavaloaei et al., 2019). More recently, they extended their computational analysis onto our published results (Ohigashi et al., 2019) and in this issue described that they confirmed their original conclusions that β5t regulates the expression of hundreds of genes in cTECs (Apavaloaei et al., 2021). Accordingly, the editors of *Cell Reports* kindly invited us to comment on their analysis and conclusions. Here, we evaluate their analysis of our data and discuss the biology of β5t and cTECs, focusing on the issues that they raised in their letter.

### β5t affects MHC class I-associated peptides without affecting the abundance of substrate proteins

In their letter, Apavaloaei et al. (2021) described that it is difficult for them to envision how β5t may affect MHC class I-associated peptide (MAP) biogenesis without affecting the abundance of MAP source proteins and transcripts, nor the myriad of biological processes regulated by proteasomes. In this context, it is relevant to note that the previous experiments showed that in comparison with an isolated preparation of β5i-containing immunoproteasomes, parallelly purified β5t-containing thymoproteasomes produce a unique set of peptides by the degradation of well-characterized standard proteins (chicken ovalbumin, yeast enolase, and bovine β-casein) *in vitro* (Sasaki et al., 2015). It was also shown that β5t-containing thymoproteasomes and β5i-containing immunoproteasomes expressed in mouse embryonic fibroblasts produce a different repertoire of MHC class I-associated peptides on the cell surface (Sasaki et al., 2015). Furthermore, a more recent study showed that human thymoproteasomes and immunoproteasomes differ in cleavage preference and peptide products quantitatively and qualitatively (Kuckelkorn et al., 2019). These results indicate that β5t affects MHC class I-associated peptides without affecting the abundance of peptide source proteins or their transcripts or the myriad of biological processes regulated by proteasomes. The β5t-containing thymoproteasomes, which carry a unique substrate-binding pocket in the β5t structure and thereby exhibit unique endopeptidase specificity in proteolysis (Murata et al., 2007; Florea et al., 2010), produce a unique set of degraded peptides and a unique repertoire of MHC class I-associated peptides without affecting the abundance of substrate proteins or their transcripts or the myriad of biological processes.

### Quality of isolated cTEC samples

In their letter, Apavaloaei et al. (2021) describe that their TEC extraction protocol and stringent gating applied during the fluorescence-activated cell sorting led to the isolation of cTEC samples with superior purity, and that the hypothesis that their observations were a product of low-quality samples can be discarded. They highlighted the fact that our cTEC preparations included cortical thymocytes, which was documented and evaluated in detail in our report (Ohigashi et al., 2019). However, it is well appreciated that the vast majority of cTECs are tightly associated with cortical thymocytes, and essentially all cTECs represent the thymic nurse cells (Venables et al., 2019). The co-purification of thymocytes with cTECs reflects this adhesive nature of the majority of cTECs and their tight association with cortical thymocytes, even after the high-purity flow cytometric isolation of cTECs based on the expression profiles of cell surface marker molecules (Nakagawa et al., 2012). This was the reason why we specifically evaluated the contribution of cortical thymocytes in RNA and protein samples extracted from isolated cTEC preparations (see Figure 2 and Figure S2 in Ohigashi et al., 2019). In parallel, flow cytometric analysis showed that our cTEC preparations are devoid of any appreciable contamination of mTECs or other thymic cells (Ohigashi et al., 2019). In contrast, Apavaloaei et al. (2019) does not show flow cytometric profiles, or any parameters for the purity, of their isolated cTEC preparations used for their transcriptomic analysis. Their study shows no results to evaluate how much their cTEC preparations were contaminated with any thymic cells, including mTECs, thymocytes, or other cells in the thymus. We wonder how they could state that their cTEC samples had “superior purity” over our cTEC samples without revealing the cell purity in their own samples.

**Figure 1.**
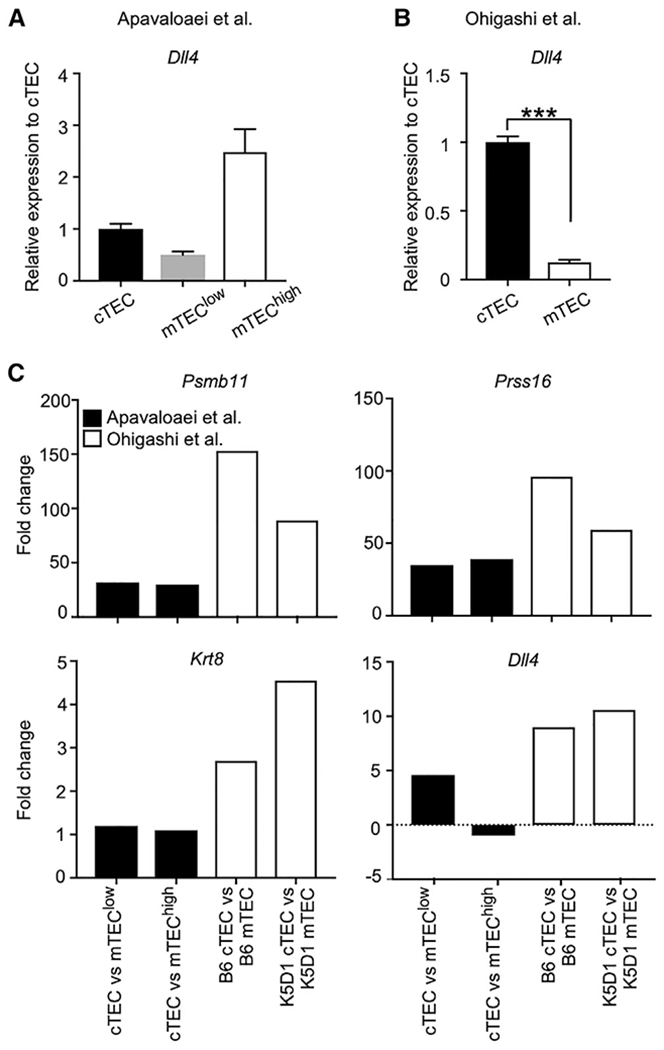
Quality of isolated cTEC samples (A and B) Abundance (means and SEMs, n = 3) of *Dll4* mRNA in transcriptomic data obtained from Apavaloaei et al. (2019) (A) and Ohigashi et al. (2019) (B). The data from the 2 studies were analyzed in parallel by using the CLC Genomics Workbench (QIAGEN). Read counts were normalized to counts in cTEC samples. ***p < 0.001 by unpaired Student’s t test. (C) Fold change in the abundance of *Psmb11, Prss16, Krt8*, and *Dll4* mRNAs between cTEC and mTEC samples in transcriptomic data by Apavaloaei et al. (2019) and Ohigashi et al. (2019).

In this regard, it is relevant to note that their transcriptomic data show that their cTEC preparations expressed lower amounts of *Dll4* mRNA than their mTEC preparations (Figure 1A; Apavaloaei et al., 2019). It is well appreciated that the majority of cTECs express higher amounts of *Dll4* mRNA, encoding DLL4 protein, than mTECs (Ribeiro et al., 2013), and this was reproduced in our transcriptomic results from our cTEC and mTEC samples (Figure 1B; Ohigashi et al., 2019). The preferential abundance of DLL4 protein in cTECs is highly relevant to the biology of cTECs and the thymic cortex for the specification and generation of T cells (Koch et al., 2008). In contrast, their transcriptomic results with the low abundance of *Dll4* in their cTEC samples (Apavaloaei et al., 2019) fail to agree with the biology of cTECs, suggesting that their cTEC preparations do not represent the majority of cTECs. Many other genes, including *Psmb11, Prss16*, and *Krt8*, which are known to be highly abundant in cTECs in comparison with mTECs, and which encode proteins that functionally and structurally characterize cTECs (Takahama et al., 2017), show a less apparent fold change in abundance between their cTEC and mTEC preparations than the fold change in abundance between our cTEC and mTEC preparations (Figure 1C). In this regard, it is possible that their cTEC preparations may be contaminated with other cells, such as mTECs. It is also possible that they might have selectively enriched a minor and atypical subpopulation of cTECs that were not tightly associated with cortical thymocytes and could be easily removed from cortical thymocytes. For example, they may have enriched the previously published minor P3 subpopulation of cTECs (Nakagawa et al., 2012). The expression profiles of functionally relevant molecules in cTECs suggest that their cTEC samples fail to represent the majority of cTECs. Therefore, the possibility that their observations are the product of the unrevealed quality of their cTEC samples should not be readily dismissed but be seriously taken into consideration.

### β5t does not regulate the expression of many genes in cTECs

**Figure 2.**
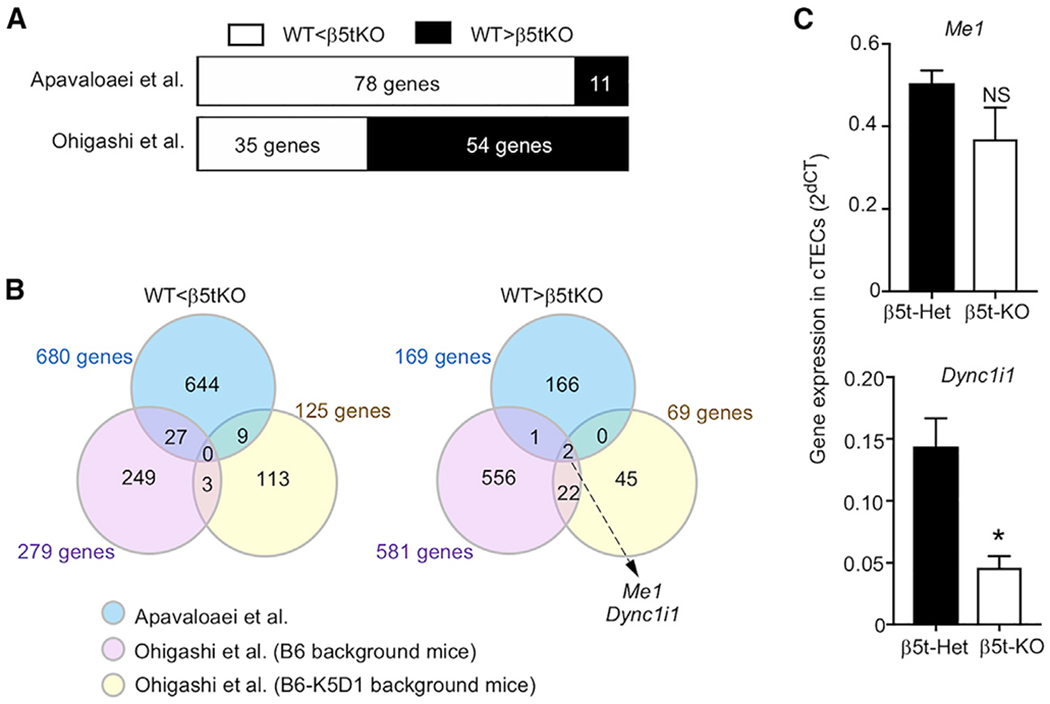
β5t does not regulate the expression of many genes in cTECs (A) Number of genes that were different in abundance between β5t-deficient cTECs and control cTECs, among 89 differentially expressed genes extracted by Apavaloaei et al. (2021). Shown are the parallel re-analyses of the results described in Apavaloaei et al. (2021, Figure 2B and Table S2) based on the data published by Apavaloaei et al. (2021) (top) and by Ohigashi et al. (2019) (bottom). (B) Venn diagrams showing the overlap among the numbers of differentially expressed genes in transcriptomic data by Apavaloaei and colleagues (blue), our B6 background data (purple), and our K5D1 background data (yellow). Results of the parallel re-analysis of the data described in Apavaloaei et al. (2021, Tables S2 and S3) are shown. (C) Quantitative RT-PCR analysis of mRNA expression levels (means and SEMs, n = 3) of indicated genes relative to *Gapdh* level in β5t-Het and β5t-KO cTECs. *p < 0.05; NS, not significant, by unpaired Student’s t test.

In their letter, Apavaloaei et al. (2021) reported the extraction of 89 genes (excluding β5t-encoding *Psmb11*) that were commonly detected in both their data and our data as differentially expressed genes between cTEC samples derived from control mice and β5t-deficient mice. They described that these 89 genes appeared to be particularly robust transcriptional targets of *Psmb11* because they were shared between the 2 datasets, supporting their conclusion that β5t regulates gene expression in cTECs (Apavaloaei et al., 2021). Of the 89 genes, 78 genes had high abundance and 11 had low abundance in cTEC samples derived from β5t-deficient mice in their data. In contrast, 35 genes had high abundance and 54 had low abundance in cTEC samples derived from β5t-deficient mice on B6 background in our data (Figure 2A). Specifically, among the 78 genes that had high abundance in β5t-deficient cTECs in their data, only 27 genes had high abundance in our data, whereas among the 11 genes that had low abundance in β5t-deficient cTECs in their data, only 3 genes had low abundance in our data (Figure 2B). By extending the analysis to include our transcriptomic data obtained from keratin 5 promoter-driven cyclin D1-transgenic (K5D1) B6 background mice (Ohigashi et al., 2019), it was further shown that between their data and our data, no genes were consistently elevated in cTECs by the loss of β5t, whereas only 2 genes, *Me1* and *Dync1i1*, in addition to β5t-encoding *Psmb11*, were reduced by the loss of β5t (Figure 2B). Moreover, we noted that the decrease in *Me1* was not reproduced in β5t-deficient cTECs in quantitative RT-PCR analysis, whereas the reduction of *Dync1i1* was reproduced in β5t-deficient cTECs relative to cTECs from control mice (Figure 2C). *Dync1i1* encodes cytoplasmic dynein 1 intermediate chain 1, although our proteomic analysis did not show that the abundance of this protein is not affected in cTECs in the presence or absence of β5t (as shown below). These results indicate that the vast majority of the β5t-dependent differentially expressed genes in cTECs described previously (Apavaloaei et al., 2019) do not show a reproducible difference in gene expression profiles in our data. Our data do not support their conclusion that β5t regulates the expression of hundreds of genes in cTECs.

It is also relevant to note that they extracted 861 genes from our transcriptomic data, which were differentially expressed between cTECs from B6 mice and cTECs from B6-β5t-deficient mice, according to their criteria, when they focused on the 89 genes shared between their dataset and ours (Apavaloaei et al., 2021), indicating that only 10% of the genes were overlapped in the 2 datasets that were independently generated in the 2 studies. This low frequency of overlapped genes is in accordance with the large difference in the quality of cTEC preparations in the two studies, as discussed above. In this context, it may be worth reiterating that their results, which were irreproducible in our data, could have been derived from their use of mice with a mixed genetic background, their use of cTECs without clarifying the purity, and/or their focus on the small difference detected by RNA sequencing analysis without confirmation by quantitative mRNA measurement (Ohigashi et al., 2019).

### No reproducible β5t-dependent alterations in adhesion and chemotaxis genes in cTECs

In their letter, Apavaloaei et al. (2021) described that the β5t-dependent differentially expressed genes extracted from our data showed an enrichment in cell adhesion genes and chemotaxis genes and that the impact of β5t on gene expression in cTECs can be replicated between their data and our data. However, as described above, the vast majority of their extracted genes, including cell adhesion-associated genes and chemotaxis-associated genes, failed to show reproducible behavior with regard to high/low abundance between their data and our data (Figure 2).

In addition, none of the specific reductions or elevations highlighted in their transcriptomic analysis of cell adhesion-associated genes and chemotaxis-associated genes, including *Cxcl12, Ccl25, Itgam, Fndc1, Col3a1, Cdh13*, and *Cldn4*, were reproduced in our results from RNA sequencing analysis and quantitative RT-PCR measurement of our cTEC samples obtained from B6 background mice (Ohigashi et al., 2019). These results indicate that our data do not reproduce the β5t-dependent alterations in adhesion and chemotaxis genes in cTECs, which they obtained by RNA sequencing analysis but did not confirm by any other methods, including quantitative RT-PCR measurement.

### No reproducible acquisition of mTEC features in β5t-deficient cTECs

Similarly, our results do not support their conclusion that cTECs from β5t-deficient mice acquire the features of mTECs. To analyze our data on this issue, Apavaloaei et al. (2021) chose to focus on only our transcriptomic data obtained from K5D1 background mice, and somehow excluded the analysis of our data from B6 background mice. They described in the extraction of 193 genes that they highlighted a shift in the expression closer to mTECs in β5t-deficient cTECs. However, as described above, the vast majority of the β5t-dependent differentially expressed genes that they extracted did not behave reproducibly in the cTEC samples in our data from B6 background mice and K5D1 background mice (Figure 2).

In addition, the cTEC genes that they reported to have reduced abundance in their cTEC samples in β5t-deficient mice, including *Enpep, Ly75, Ctsl, Prss16, Cxcl12, Ccl25*, and *Dll4* (Apavaloaei et al., 2019), did not show reduced abundance in our data from the RNA sequencing analysis and quantitative RT-PCR analysis (Ohigashi et al., 2019). Thus, our data do not support their conclusion that β5t-deficient cTECs acquire mTEC features or β5t regulates gene expression in cTECs.

### Proteomic impact on proteasome subunit composition in β5t-deficient cTECs

**Figure 3.**
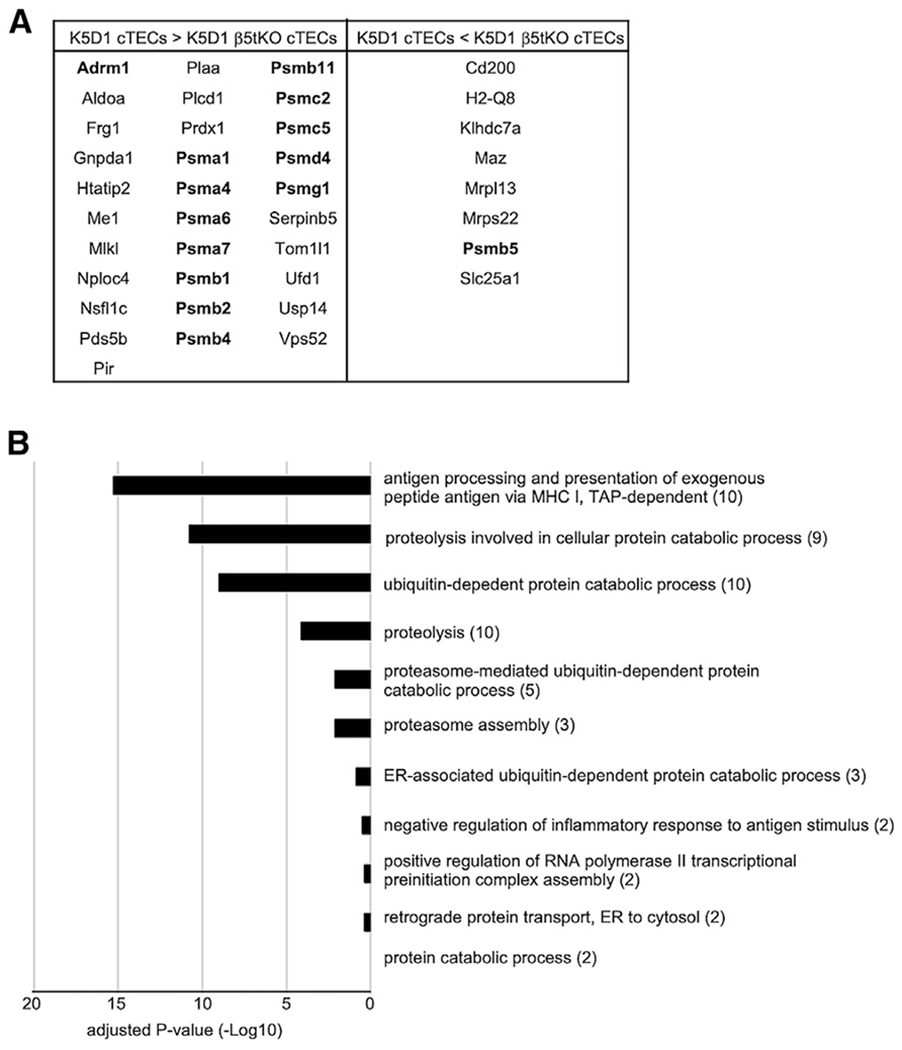
Proteomic impact on proteasome subunit composition in β5t-deficient cTECs (A) Thirty-nine proteins that were significantly (p < 0.05) altered in abundance between K5D1 cTECs and K5D1-β5t-deficient cTECs in our TMT-based quantification, among 415 differentially expressed proteins that Apavaloaei et al. (2021) extracted from our label-free proteomic analysis. Eight core particle components and six regulatory particle components of proteasomes are highlighted in bold letters. (B) Gene Ontology enrichment analysis of proteins shown in (A). The numbers in parentheses indicate the number of categorized proteins.

They finally extended their computational analysis onto our proteomic data and described that 415 proteins were differentially expressed in cTECs between control K5D1 mice and β5t-deficient K5D1 mice. They described that several transcription factors, cytoskeleton proteins, and adhesion molecules are included in those differentially expressed proteins (Apavaloaei et al., 2021). However, they chose to exclusively analyze our mass spectrometry data derived from two experiments of liquid chromatography-tandem mass spectrometric (LC-MS/MS)-based label-free analysis, which we carried out to back up our proteomic observations from tandem mass tag (TMT)-labeled LC-MS/MS quantification. They somehow excluded the analysis of our main proteomic results obtained from the TMT-labeled LC-MS/MS quantification of K5D1 cTECs in quadruplicate and K5D1-β5t-deficient cTECs in triplicate. It is well appreciated that the TMT labeling method is superior to the label-free method in the precision of proteomic quantification, especially of low abundant proteins (Hogrebe et al., 2018; Pappireddi et al., 2019). Our results from TMT-labeled quantification showed that among the 415 differentially abundant proteins that they extracted from our label-free analysis, only 39 proteins showed consistently increased or reduced abundance between K5D1 cTECs and K5D1-β5t-deficient cTECs with a significant (p < 0.05) difference (Figure 3A). Thus, the majority of proteins that they extracted from our label-free analysis (91%, 376 of 415 proteins) showed no reproducible difference in our TMT-labeled quantification.

Nonetheless, among the 39 proteins that were consistently extracted from our label-free and TMT-labeled LC-MS/MS analysis, 14 proteins represented core particle components and regulatory particle components of the proteasomes (Figure 3A), as we reported in our previous article (Ohigashi et al., 2019). In addition, the Gene Ontology enrichment analysis of these 39 proteins showed that the cTEC proteins affected by β5t deficiency are highly enriched with proteasome components and proteasome-related proteins (Figure 3B), in agreement with our previous conclusion that the presence or absence of β5t specifically affects the composition of proteasome subunits in cTECs (Ohigashi et al., 2019).

Our analysis confirms that the presence or absence of β5t specifically affects proteasome subunit composition in cTECs, without pervasively affecting the abundance of various mRNAs and proteins in cTECs, and without affecting the abundance of a variety of molecules, including transcription factors, cytoskeleton proteins, and adhesion molecules.

## DISCUSSION

Apavaloaei et al. (2021) analyzed our transcriptomic data and described that they confirmed their original conclusion that β5t regulates the expression of hundreds of genes in cTECs. They extracted 89 differentially expressed genes by the analysis of a partial set of our data. However, our examination of those 89 genes in all sets of our RNA sequencing data along with confirmation by quantitative RT-PCR analysis clarified that only one gene, *Dync1i1*, in our transcriptomic data meets their definition of β5t-dependent altered abundance in cTECs. However, our proteomic data failed to detect *Dync1i1*-encoded protein in the list of cTEC proteins that were significantly altered in abundance by the β5t deficiency. Thus, our data failed to verify their conclusion that β5t regulates the expression in cTECs of many genes, including the genes involved in cell adhesion and chemotaxis and including the genes associated with mTEC features.

Apavaloaei et al. (2021) also analyzed our proteomic data and described that 415 proteins were differentially expressed between control cTECs and β5t-deficient cTECs. Again, they chose to analyze a small fraction of our dataset, exclusively focusing on our label-free LC-MS/MS data. However, our examination of those proteins in all sets of our proteomic data, including the TMT-labeled LC-MS/MS data and the label-free LC-MS/MS data, clarified that among those 415 proteins, only 39 proteins showed consistently high or low abundance between control cTECs and β5t-deficient cTECs. Those 39 proteins are highly enriched with proteasome components and proteasome-related proteins, in agreement with our previous conclusion that the presence or absence of β5t specifically affects the composition of proteasome subunits in cTECs. Thus, our data contradict their conclusion that a variety of proteins, including a group of transcription factors and cell adhesion molecules, differ in the abundance in β5t-deficient cTECs.

Accordingly, their analysis of our results was always partial and biased, as they focused only on a fraction of our data and excluded the rest of parallelly published data (i.e., only focusing on either B6 data or K5D1 data while excluding the other data in transcriptomic analysis, and only focusing on label-free LC-MS/MS data while excluding TMT-labeled LC-MS/MS data in proteomic analysis). In addition, their differential expression plainly included the genes that have elevated abundance in one dataset and reduced abundance in another dataset, and failed to take into account biological reproducibility in the different abundance of the molecules. Consequently, our re-examination of their extracted molecules in all of our datasets disproves their conclusions.

In our previous report, we showed results indicating that we did not detect any increase in the abundance of β-catenin proteins in β5t-deficient cTECs. Despite that, Apavaloaei et al. (2019) reported that β5t regulates the expression of hundreds of cTEC genes via the Wnt signaling pathway. We also showed that neither the number of MHC class I^high^ CD69^low^ CCR7^high^ TCRβ^high^ CD4^+^CD8^−^ thymocytes nor the expression of oxidative stress genes in TCRβ^high^ CD4^+^CD8^−^ thymocytes was increased in β5t-deficient mice, despite the descriptions in their report. Thus, our results, including the previously published flow cytometric results and the transcriptomic and proteomic results discussed in this article, do not support their conclusions that β5t regulates the expression of hundreds of cTEC genes via the Wnt signaling pathway and has pervasive effects on thymocytes, including CD4^+^CD8^−^ thymocytes, by causing oxidative stress. Instead, our re-analysis here reconfirms the conclusion that β5t has a highly specific impact on proteasome subunit composition in cTECs.

The highly specific impact of β5t on proteasome subunit composition in cTECs supports the possibility that the β5t-containing thymoproteasome optimizes CD8^+^ T cell development through the proteasomal production of MHC class I-associated unique self-peptides in cTECs. We recently showed that the thymoproteasome shapes the T cell receptor (TCR) repertoire directly in cortical thymocytes before migration to the thymic medulla and independent of apoptosis-mediated negative selection (Ohigashi et al., 2021). The thymoproteasome may contribute to the preferential production of MHC class I-associated self-peptides that carry structural advantages to interact with TCR structures that are positively selected into CD8^+^ T cells. We think that the biochemical identification of β5t-dependent MHC class I-associated peptide sequences freshly isolated from mouse cTECs will benefit our understanding of the fundamental basis for the thymus-dependent positive selection of T cells.

## STAR★METHODS

### RESOURCE AVAILABILITY

#### Lead contact

Requests for further information may be directed to and will be fulfilled by the lead contact Yousuke Takahama (yousuke.takahama@nih.gov).

#### Materials availability

This study did not generate new unique reagents.

#### Data and code availability

This study used datasets published by (Apavaloaei et al. (2019, 2021). Their RNA sequencing data were downloaded from Gene Expression Omnibus database (https://www.ncbi.nlm.nih.gov/geo/) under accession numbers GSE107535 and GSE107536. The differentially expressed genes and proteins according to their analysis are shown in Tables S2, S3, and S4 in Apavaloaei et al. (2021). Our RNA sequencing data are available from The DNA Data Bank of Japan database (https://www.ddbj.nig.ac.jp) with the accession number DRA008167. MS proteomic data from TMT-based quantification and label-free quantification are available from ProteomeXchange Consortium via jPOST partner repository (https://repository.jpostdb.org) with the dataset identifiers PXD013132 and PXD013133.

### EXPERIMENTAL MODEL AND SUBJECT DETAILS

#### Mice

β5t-deficient mice were described previously (Murata et al., 2007) and backcrossed to B6 background. Male mice were used for experiments at 8 to 10 weeks old. Mice were housed on a 12-hour light-dark cycle in climate-controlled, pathogen-free barrier facilities. Mouse experiments were performed with consent from the Animal Experimentation Committee of the University of Tokushima (T2019-62) and from the Animal Care and Use Committee of the National Cancer Institute (ASP 18-431 and EIB-076-2).

### METHOD DETAILS

#### Isolation of cTECs

Minced thymuses were digested with 0.5 unit/mL Liberase TM (Roche) in the presence of 0.02% DNase I (Roche). CD45^−^ cells were enriched with magnetic-bead-conjugated anti-CD45 antibody (Miltenyi Biotec). CD45^+^ cell-depleted single-cell suspensions were stained for the expression of EpCAM (CD326, BioLegend, clone G8.8), CD45 (BioLegend, clone 30-F11), Ly51 (CD249, BioLegend, clone 6C3), and for the reactivity with UEA1 (Vector Laboratories). Cell sorting of cTECs were performed on FACSAria II (BD Biosciences).

#### Quantitative RT-PCR analysis

RNA extraction from isolated cTECs was performed using RNeasy Plus Micro Kit (QIAGEN). Total cellular RNA was reverse-transcribed (RT) with PrimeScript Reverse Transcriptase (TaKaRa). Quantitative real-time polymerase chain reaction (PCR) was performed using SYBR Premix Ex Taq (TaKaRa) and a StepOnePlus Real-Time PCR System (Applied Biosystems). The amplified products were confirmed to be single bands by gel electrophoresis.

#### RNA sequencing data processing

Data were analyzed by using CLC Genomics Workbench 11 (QIAGEN) with default parameters.

#### Gene Ontology term analysis

Gene ontology for transcriptomic and proteomic data were analyzed by using DAVID Bioinformatics Resources 6.8 (https://david.ncifcrf.gov).

### QUANTIFICATION AND STATISTICAL ANALYSIS

Statistical analysis was carried out using GraphPad Prism 7 software. Statistical significance was assessed using the two-tailed unpaired Student’s t test with Welch’s correction for unequal variances.

## Figures and Tables

**Table:** KEY RESOURCES TABLE

REAGENT or RESOURCE	SOURCE	IDENTIFIER
**Antibodies**		
CD45 MicroBeads, mouse antibody	Miltenyi Biotec	Cat# 130-052-301; RRID:AB_2877061
PE/Cy5 anti-mouse CD45 antibody (30F-11)	BioLegend	Cat# 103110; RRID:AB_312975
PE/Cy7 anti-mouse CD326 (EpCAM) antibody (G8.8)	BioLegend	Cat# 118216; RRID:AB_1236471
Alexa Fluor 647 anti-mouse Ly51 antibody	BioLegend	Cat# 108312; RRID:AB_2099613
Biotin Ulex europaeus agglutinin I (UEA I)	Vector Laboratories	Cat# B-1065; RRID:AB_2336766
Streptavidin APC-eFluor 780	Invitrogen	Cat# 47-4317-82; RRID:AB_10366688
**Critical commercial assays**		
RNeasy Plus Micro Kit	QIAGEN	Cat# 74034
**Deposited data**		
Apavaloaei et al. RNA sequencing data	Gene expression Omnibus database (Apavaloaei et al., 2019)	GSE107535 GSE107536
Ohigashi et al. RNA sequencing data	The DNA Data Bank of Japan (Ohigashi et al., 2019)	DRA008167
Ohigashi et al. MS proteomic data	ProteomeXchange Consortium (Ohigashi et al., 2019)	PXD013132 PXD013133
**Experimental models: Organisms/strains**		
Mouse: β5t ^−/−^	Murata et al., 2007	N/A
**Oligonucleotides**		
PCR Primer for Me1 Forward: 5′-CCCTGAGTATGACGCCTTCC-3′	This paper	N/A
PCR Primer for Me1 Reverse: 5′-GCAACAGACGCTGTTCCTTG-3′	This paper	N/A
PCR Primer for Dync1i1 Forward: 5′-ACAACAAGCCGCTCTACTCC-3′	This paper	N/A
PCR Primer for Dync1i1 Reverse: 5′-AACTTCCTTGCCACCCTGTG -3′	This paper	N/A
PCR Primer for Gapdh Forward: 5′-CCGGTGCTGAGTATGTCGTG-3′	This paper	N/A
PCR Primer for Gapdh Reverse: 5′-CAGTCTTCTGGGTGGCAGTG -3′	This paper	N/A
**Software and algorithms**		
GrapPad Prism 7	GrapPad	RRID:SCR_002798
CLC Genomics Workbench	QIAGEN	RRID:SCR_011853
DAVID	Leidos Biomedical Research, Inc	RRID:SCR_001881

## Highlights

- β5t specifically affects the abundance of proteasome subunit composition in cTECs
- β5t does not reproducibly affect the abundance of hundreds of cTEC genes
